# The Dynamics of Coalition Formation on Complex Networks

**DOI:** 10.1038/srep13386

**Published:** 2015-08-25

**Authors:** S. Auer, J. Heitzig, U. Kornek, E. Schöll, J. Kurths

**Affiliations:** 1 Institute for Theoretical Physics, Technische Universität Berlin—Hardenbergstr. 36, 10623 Berlin, Germany, EU; 2 Potsdam Institute for Climate Impact Research—P.O. Box 60 12 03, 14412 Potsdam, Germany, EU; 3 Department of Physics, Humboldt University—Newtonstr. 15, 12489 Berlin, Germany, EU; 4 Institute for Complex Systems and Mathematical Biology, University of Aberdeen—Aberdeen AB24 3FX, UK, EU; 5 Department of Control Theory, Nizhny Novgorod State University—Gagarin Avenue 23, 606950 Nizhny Novgorod, Russia; 6 Institute of Applied Physics of the Russian Academy of Sciences—Uljanov str. 46, 603950 Nizhny Novgorod, Russia

## Abstract

Complex networks describe the structure of many socio-economic systems. However, in studies of decision-making processes the evolution of the underlying social relations are disregarded. In this report, we aim to understand the formation of self-organizing domains of cooperation (“coalitions”) on an acquaintance network. We include both the network’s influence on the formation of coalitions and vice versa how the network adapts to the current coalition structure, thus forming a social feedback loop. We increase complexity from simple opinion adaptation processes studied in earlier research to more complex decision-making determined by costs and benefits, and from bilateral to multilateral cooperation. We show how phase transitions emerge from such coevolutionary dynamics, which can be interpreted as processes of great transformations. If the network adaptation rate is high, the social dynamics prevent the formation of a grand coalition and therefore full cooperation. We find some empirical support for our main results: Our model develops a bimodal coalition size distribution over time similar to those found in social structures. Our detection and distinguishing of phase transitions may be exemplary for other models of socio-economic systems with low agent numbers and therefore strong finite-size effects.

Statistical physics provides a powerful means to conceptually study mechanisms of socio-economic systems and their associated transformations such as market restructuring, social upheavals and revolutions. Many socio-economic systems exhibit network structures^1^, and a number of studies show how network structures influence behaviour such as bilateral cooperation^2^. Much less work is done on the reverse effect that the network structure in turn adapts to behaviour^3^,^4^,^5^. While both processes are interesting in themselves, in the context of opinion dynamics it is actually the feedback loop of both network adaptation and dynamics on the network which leads to the most interesting nonlinear effects. E.g., the seminal work of Holme^6^ presents a model in which a phase transition occurs that can be interpreted as a great transformation.

In this report, we transfer the methods of Holme^6^ from local social dynamics to a more complex form of mesoscopic social self-organization, namely that of multilateral cooperation (here called *coalitions*), whose interaction with network structures has not been studied before. In particular, we present a model of the coevolution of an adaptive network representing social acquaintance and a coalition structure which is a partition of nodes into coalitions of arbitrary size representing multilateral cooperation (Fig. 1). Instead of an exogenously given number of opinion groups as previously studied in the literature, the number of coalitions in our model evolves endogenosly as a process of self-organization from the boundedly rational behavior of the agents. Our model can be applied to socio-economic environments where cooperation promises economic or social advantages, and to study such diverse subjects as firm size distributions, fish cohorts, and political parties. Our methods to detect phase transitions are especially applicable to small real-world systems. However, in our case low sample sizes do not necessarily indicate interaction processes with a low number of people since each agent might be composed of many individuals, already. A common economic situation for which cooperation is critical is the use of a common pool resource. It leads to nontrivial coalition formation dynamics because agents not only have an incentive to form a coalition but also to leave a coalition in order to profit from the efforts of the remaining coalition. Since one of the major current economic challenges, the transition to a low-carbon economy, is closely related to several common pool resources like the atmosphere and renewable energy sources, we focus on the application of our model to common pool resources in this article.

## Results

In our model, each coalition rationally decides how much of the resource to exploit and gets a corresponding payoff that depends on all coalitions’ sizes and decisions. On this basis, individual agents rationally decide to form new coalitions with their acquaintances or merge or leave existing coalitions. Finally, they may also form new acquaintance links to members of their own coalition or break existing ones to members of other coalitions. The main control parameter in our model is the relative speed of acquaintance adaptation vs coalition formation, the adaptation rate *ϕ*, and the main feature of the resulting dynamics is the distribution of coalition sizes that evolves as an equilibrium over time.

**Figure 2:**
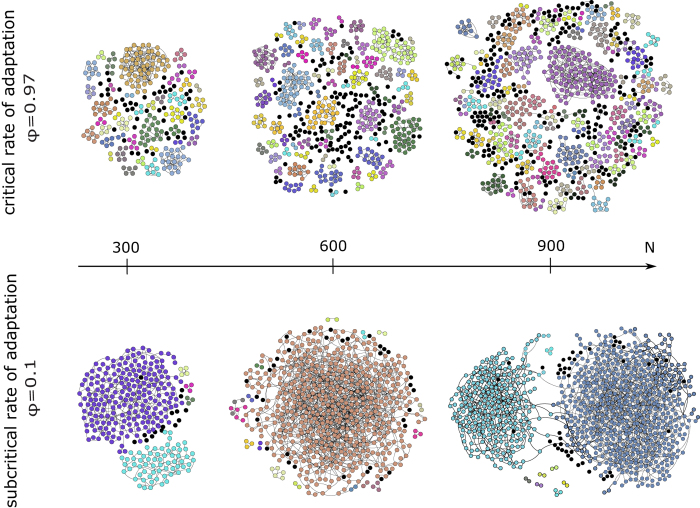
Acquaintance network with coalition structure (each color represents one coalition, black dots are singleton coalitions) for varying system size (columns: *N* = 300, *N* = 600 and *N* = 900) and adaptation rate (rows: *ϕ* = 0.97 and *ϕ* = 0.1). Note that some of the smaller network components consist of more than one coalition. Each network is the equilibrium result of one model run.

**Figure 3:**
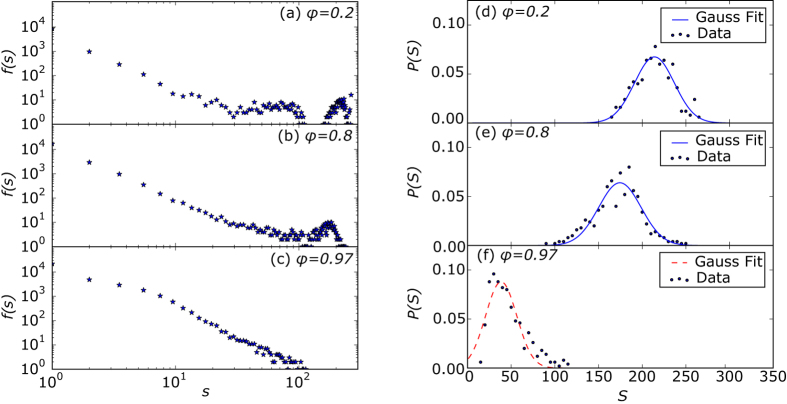
Left (**a**–**c**) log-log plot of frequency distribution of *all* coalition sizes *s* and right (**d**–**f**) histograms *P*(*S*) of *maximum* coalition size *S* in the consensus state for *ϕ* = 0.2, *ϕ* = 0.8 and *ϕ* = 0.97, respectively. *N* = 300 and 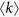 = 3 (for 500 model runs).

For the case of agents exploiting a common pool resource^7^, we find a second order phase transition when adaptation versus coalition formation crosses its critical value, *ϕ* = *ϕ*_*c*_. For subcritical adaptation rates (see Methods for the description of the model and parameters), the coalition structure is dominated by very few macroscopic or even near-global coalitions. This leads to a peculiarly multimodal size distribution that can also be observed in various real-world systems^8^,^9^,^10^,^11^,^12^,^13^, not only in socio-economic contexts but also in purely physical systems such as droplets^14^. In contrast, at the critical adaptation rate, a more heterogeneous but power-law-tailed size distribution with much smaller maximal coalitions emerges (see Figs 2 and 3).

### Change in Coalition Size Distributions

We see significant changes in the distribution from a few macroscopic coalitions to complex multimodal distributions, when the adaptation rate *ϕ* is changed. There are two extremes. For *ϕ* = 0 the dynamics are purely based on coalition formation and hence coalition sizes approach the initial component sizes. In contrast, for *ϕ* = 1 only network adaptation takes place: starting with a coalition structure of only singletons, this parameter setting immediately converges without any further changes taking place; from the beginning, there are no coalition partners to link with. For small *ϕ* the distribution has peculiar features: a linearly decreasing frequency for small coalition sizes *s* and one or two local maxima for larger coalition sizes. Its multimodal nature emerges endogenously from the nonlinear dynamics of coalition formation. As a matter of fact, from empirical observations, multimodal distributions of social structures are well known, e.g. multimodal size distributions have been found in growth patterns of fish cohorts^8^,^9^ and droplet sizes^14^, in human communication^10^ and in firm and city size distributions of developing countries^11^,^12^,^13^.

**Figure 4:**
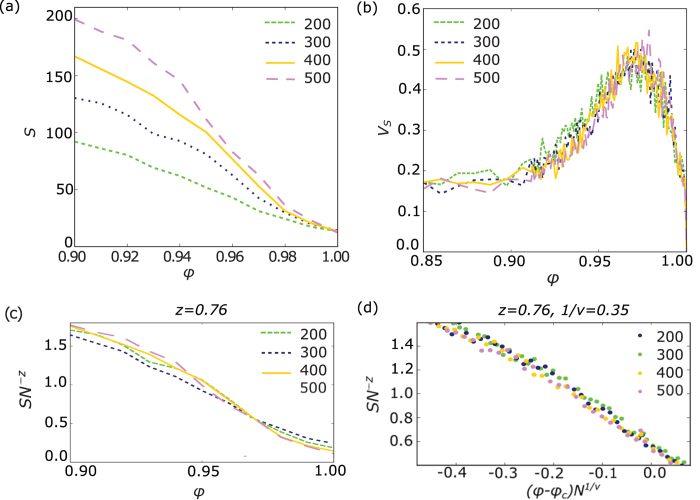
Plot of (**a**) order parameter *S*, (**b**) coefficient of variation *V*_*S*_ and (**c**) *S* scaled with *N*^−*z*^ over control parameter *ϕ* for different agent numbers *N*. (**d**) Data collapse close to the critical point *ϕ*_*c*_. *S* scaled with *N*^−*z*^ over (*ϕ* − *ϕ*_*c*_) scaled with *N*^*ν*^. All variables are averaged over 100 model runs.

For a typical size of coalition forming systems, *N* = 300 nodes, a look at the distribution of *s* in steps of Δ*ϕ* = 0.1 initially does not reveal any interesting artifacts such as power-laws. However, at values of *ϕ* close to one, the local maxima at the tails disappear, and for a critical adaptation rate *ϕ*_*c *_≈ 0.97 coalition sizes show a power-law tail (Fig. 3c). The reason for such a high critical value are the more macroscopic effects of the coalition formation process as compared to the network adaptation process; it may involve hundreds of agents at once whereas network adaptation only affects three agents at a time (see Methods). In Fig. 4a, a higher resolution plot of maximum coalition size *S* vs. *φ* shows a turning point of this order parameter^15^, something we expect for a second-order phase transition. It is not very distinctive but this is expected due to finite size effects^16^. Samples of up to 500 nodes are rather small for statistical physics and an exponential progression of system sizes would be more revealing as network distances scale slowly with *N*. Still, we chose a linear progression in *N* because the coalition formation process causes high computational costs with rising *N* and agent numbers of up to several hundreds are quite realistic for many socio-economic systems^17^. Nevertheless, phase transitions appear, only the accompanying singularities are washed out or smoothed^18^ due to finite-size effects. Also, we expect only small finite-size corrections^19^ to critical scaling with an exponential progression to larger *N* which are insignificant in the context of socio-economic modeling. Thus, the detailed analysis of finite-size phase transitions may be of great use for further models in this context.

### Second-order Phase Transition

From these plots alone, the type of phase transition (first- or second-order) is hard to identify because a finite sample size will give both first- and second-order transitions a similar appearance. However, the type of transition is revealed by the probability density function of the order parameter *S*. For a varying control parameter *ϕ*, first-order transitions have two peaks at fixed position with changing height^20^. Whereas for a second-order transition, a Gaussian peak continuously changes its position—the maximum and mean values of *S* are moving to smaller values for increasing *ϕ*^18^ until the Gaussian converts to a heavy-tailed function at the critical point. In our case, for *ϕ* = 0.2 and *ϕ* = 0.8, the Gaussian curve fits relatively well, but for *ϕ* = 0.97 there is an obvious mismatch. For Fig. 3d with *χ*^2^ = 21.7, and for Fig. 3e with *χ*^2^ = 29.6, the Chi-squared test statistics is below the critical quantile, 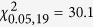. This means that on the level 5% we cannot reject the hypothesis of a normal distribution. For Fig. 3f, *χ*^2^ = 242.2 exceeds the critical quantile 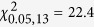. A long tail appears, featuring bigger coalition sizes that cannot be explained by a Gaussian distribution^18^. Both arguments underline the assumption of a continuous phase transition.

### Quantification of the Scaling Relation

Via the maximum of the coefficient of variation of *S*, *V*_*S*_, it is possible to identify the critical region (Fig. 4b). According to scaling theory, for different agent numbers, *V*_*S*_ should peak at about the same value of *ϕ*, only slightly shifted from the critical point by *const*. ⋅ *N*^−1/*ν*^, where *ν* is a critical exponent^21^. In the region close to the expected critical point *ϕ*_*c*_, we have estimated *V*_*S*_ for several agent numbers and indeed all maxima appear approximately at *ϕ* = 0.97, in accordance with our earlier estimate. With this knowledge, it is possible to quantitatively grasp the critical dynamics. To determine the critical exponents, it is important to recall the classical scaling relation^21^ and apply it to our model case, where *ϕ* takes the role of temperature and *S* the role of magnetization (see Methods for further explanation):


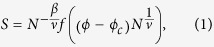


where *β* is another critical exponent. At first, it should be possible to find a scaling exponent *z* for which *SN*^z^ intersects for all agent numbers in a single point (*ϕ*_*c*_, *f*(0))^22^. Therefore, we vary *z* until a value *ϕ*_*c*_ is found where all curves cross. This is the case for *z* ≈ 0.76. At this point *z* = *β*/*ν*. In Fig. 4c, the result of successfully scaling the order parameter is shown. After that, scaling (*ϕ* − *ϕ*_*c*_) by the factor 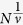 will lead to a data collapse in a region closely drawn around the critical point *ϕ*_*c*_^22^. This way, in Fig. 4d the critical exponent *ν* was found to be approximately *ν* = (0.35)^−1^.

## Discussion

What can we infer from these results? If the acquaintance network in our coalition formation model adapts only slowly to the coalition structure, the formation of a grand coalition is most probable. Only for really high adaptivity, a fast transition to a heterogeneous coalition structure appears because then the effect of coalition formation is suppressed by a permanent rewiring of the acquaintance links. Before it is even possible to find a neighbor coalition to unite with, at some earlier stage the link to this coalition was already removed. If adaptivity represents some kind of social punishment (deprivation of social contact between agents from different coalitions) then in this case punishment would actually be counterproductive. At high frequency it leads to the isolation of a high number of small and midsize coalitions forming independent network components. However, full cooperation provides the highest benefits to all agents in many socio-economic situations, including the common pool context of our study. From an outside perspective, e.g. consumers facing an oligopoly, it may however be desirable to keep coalitions (cartels) small. Of course, this phenomenon of coalition isolation is caused by our assumption that the total number of acquaintances stays constant over time which has been argued to be approximately realistic in social relations^1^. As this assumption has such large effect on the model outcome and implications, it would be most interesting to study different scenarios in future work. In the context of contemporary issues, our findings can be used to support transformation processes by fostering the persistence of social networks by lowering *ϕ*. Both our model and real-world systems may undergo non-equilibrium phase transitions (in equilibrium physics an isolated system maximizes its entropy whereas non-equilibrium phase transition are driven by an external force, e.g. a heat bath, or control parameter^23^) and therefore the investigation of socio-economic transformations can profit from conceptual models of decision-making processes. Less realistic is the investigation of agents in a fixed state after model convergence. However, after only a few time steps we observe the same basic appearance of coalition size distributions. E.g. in the subcritical case, we see a multimodal distribution with the local maxima migrating to greater values with evolving time (similar to^14^). Still, this aspect is part of ongoing work.

In our model, we have increased the level of complexity from simple opinion adaptation processes from earlier research to more complex decision making determined by costs and benefits, and from local social interaction to mesoscopic cooperation. Our approach gets support from empirical data. In our model, the fat-tailed and bimodal coalition size distributions develop over time, they are model-inherent. The distributions resulting from such processes of self-organization deliver measurable quantities to study such transformation processes. Future work should vary the payoff sub-model in order to represent different archetypical socio-economic situations than the common-pool setting. One may then compare them with firm size distributions^12^,^13^,^17^ from different economic sectors to identify the drivers of firm size growth. Other cases of multimodality in social systems were found in city size distributions of developing countries^11^ and fish cohorts^8^,^9^ possibly resulting from cooperative phenomena. However, even natural processes such as droplet growth give a similar picture: a power-law distribution for small droplets and a maximum for larger ones^14^. For the study of specific real-world systems, it might however be necessary to model heterogeneous agents. But most importantly, the observation of phase transitions with respect to network adaptivity in our model encourages follow-up work on the role that the relative speed of processes in social feedback loops has for transformation process.

## Methods

We start with an Erdös-Rényi random acquaintance network and a coalition structure composed of one singleton “coalition” per agent (coalitions of size 1), representing no initial cooperation. Coalitions are collective decision-makers and the members of a coalition act as one player and therefore, each agent can only be a member of one coalition. Over time, any number of coexisting but disjoint coalitions may emerge, each of which has to be a connected set of nodes in the network, i.e., formally a coalition structure is a partition of the network nodes into connected sets^24^. Each coalition *i* generates a payoff flow Π_*x*_ for each of its members *x* given by


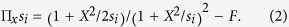


In this, *s*_*i*_ is the size of coalition *i*, *F* a parameter representing the fixed costs of maintaining a coalition, and *X* the solution to the equation


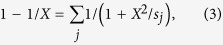


where the sum is over all coalitions *j* (see the Supporting Information, SI, for an economic derivation of this equation from a common pool resource exploitation game). In each time-step of the model, either of two processes occur:

1. With probability *ϕ*, the network adapts to the coalition structure by rewiring one cross-coalitional link of a randomly chosen agent *x* to another randomly chosen member of *x*’s coalition (unless *x* is already linked to all her coalition members). This keeps the total number of links constant which is approximately true for many real-world social systems^25^.
2. Otherwise, i.e., with probability 1 − *ϕ*, a randomly chosen agent *x* may change the coalition structure. The agent may either
  - leave her coalition (in which case the rest of the coalition splits up into its connected components),
  - merge her coalition with any combination of her neighbors’ coalitions (in which case this merger must be profitable to all affected nodes in terms of the underlying payoff model),
  - or do nothing, depending on which of these moves results in the largest next time-step’s payoff for *x*.

Note that the amount of change caused by one instance of process 1 is restricted to only three nodes, while process 2 typically affects a much larger number of nodes in one step, especially when the involved coalitions are already meso- or macroscopic.

The model has converged when no agents are able to rewire their links or find it profitable to change the coalition structure any longer. In the corresponding steady state there may still be several coalitions in each connected component of the network (see Fig. 2). Thus, the order parameter defining order and disorder in this socio-economic context is not the network component size but the size of the largest coalition, *S*. If we imagine assigning different coalitions to different spin directions, it is possible to draw an analogy to the magnetic spin model^20^. If all nodes are singletons, there are *N* different coalitions whose sizes does not exceed 1 (hence, *S* = 1). In the analogy, all spins would be pointing into different directions averaging out to a macroscopic magnetization of zero. The other extreme would be the state of a grand coalition where *S* = *N*. Here, all spins would be pointing into the same direction resulting in a non-zero magnetization (the ferromagnetic state). The transition from one state of the order parameter to the other can be of first or second order. In our model, without network adaptation (for *ϕ* = 0) the largest coalition converges to a size *S* of the order of *N*. With increasing *ϕ* the coalition formation process is increasingly disturbed and *S* decreases. Therefore, the adaptation rate *ϕ* is the natural choice for the control parameter.

From the feedback loop between coalition and network structure we expect the dynamics of this model to be highly non-linear. We study these dynamics with varying control parameter *ϕ*, in particular the occurrence of non-equilibrium phase transitions. Phase transitions can be identified and characterized with the help of scaling theory which states characteristic system variables (order parameters) to be power-law distributed at the critical value of the control parameter^21^,^23^. A visualization of the coalition structure for different system sizes gives a first insight (Fig. 2). At the critical point, system patterns should not substantially change for different system sizes. As a quantification we accompany this graphical hint of finite-size scaling with the frequency distribution of all coalition sizes, *s*, that remain after the model has converged (Fig. 3a–c), which may take up to 10^6^ time steps.

## Additional Information

**How to cite this article**: Auer, S. *et al.* The Dynamics of Coalition Formation on Complex Networks. *Sci. Rep.* **5**, 13386; doi: 10.1038/srep13386 (2015).

## Figures and Tables

**Figure 1:**
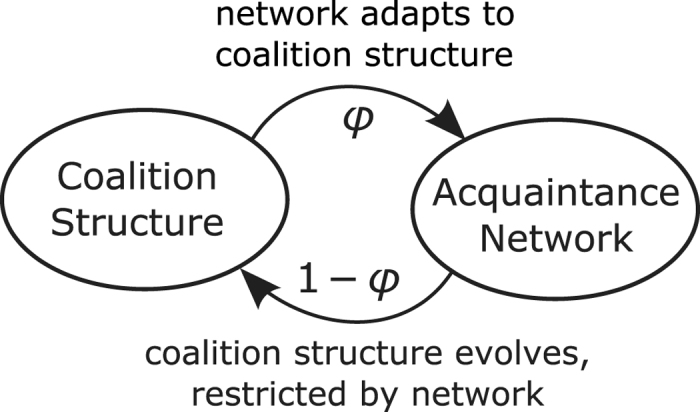
Scheme of coevolution. In each period, either one random cross-coalition links is replaced by an intra-coalitional link (adaptation with rate *ϕ*) or some random agent changes the coalition structure (coalition formation with rate 1 − *ϕ*), where each two members of a coalition must be connected by a path in the network.
