# Erratum: The Dynamics of Coalition Formation on Complex Networks

**DOI:** 10.1038/srep46983

**Published:** 2018-05-11

**Authors:** S. Auer, J. Heitzig, U. Kornek, E. Schöll, J. Kurths

Scientific Reports 5: Article number: 1338610.1038/srep13386; published online: 08 25 2015; updated: 08 11 2018

In the original version of this Article, the Supplementary Information file containing the derivation for individual payoffs was omitted. This error has now been corrected in the PDF and HTML versions of the Article.

